# Ultrasound Guided Laparoscopic Port Placement in a Patient with Congenital Anomaly of IVC Undergoing Sleeve Gastrectomy

**DOI:** 10.1007/s11695-019-04379-1

**Published:** 2020-01-07

**Authors:** Hatem Al-Saadi, Sona Singh, Kanagaraj Marimuthu, Alistair Sharples, Nagammapudur Balaji, Biju Thomas, Vittal Rao

**Affiliations:** Consultant Upper GI/Bariatric Surgeon, North Midlands Institute of Metabolic and Bariatric Surgery, University Hospital of North Midlands, Stoke-on-Trent, Staffordshire UK

**Keywords:** Ultrasound, IVC Anomaly

## Abstract

We report a patient with obesity who underwent laparoscopic sleeve gastrectomy after pre-operative ultrasound mark up to enable safe port insertion due to presence of venous collaterals in the abdominal wall as a result of congenial IVC anomaly. This patient was falsely presumed to have NASH cirrhosis. Detailed preoperative workup ruled this out and led to the discovery of congenital IVC anomaly as the cause of engorged blood vessels in the anterior abdominal wall. On table ultrasound mark up of safe sites for port insertion enabled a safe laparosocpic sleeve gastrectomy on this patient.

## Introduction

Patients with obesity can have NASH with cirrhosis as one of the common comorbidities. They may present with prominent blood vessels in the abdominal wall if they have established portal hypertension (HT). We present an interesting case where the patient had prominent blood vessels in the abdominal wall with no associated portal HT. Investigative work-up revealed an atretic inferior vena cava (IVC) with collaterals as the cause of his engorged vessels in the abdominal wall. Preoperative ultrasound marking and guided port insertion enabled the patient to undergo a safe sleeve gastrectomy.

## Case Report

A 52-year-old gentleman presented to the bariatric clinic for consideration of bariatric surgery. He had a BMI of 53.8 with a body weight of 160 kg. He had significant comorbidities which included NASH, NIDDM, AF, OSA, and history of recurrent DVT.

He had history of recurrent DVT and chronic venous insufficiency of both legs resulting in chronic venous ulcers since the age of 20. Clinical examination revealed distended veins in the anterior abdominal wall and both flanks and was presumed to have portal HT secondary to NASH Cirrhosis. However, an upper GI endoscopy ruled out varices, and an USS abdomen revealed NASH with no evidence of cirrhosis and a normal PV flow.

**Fig. 1:**
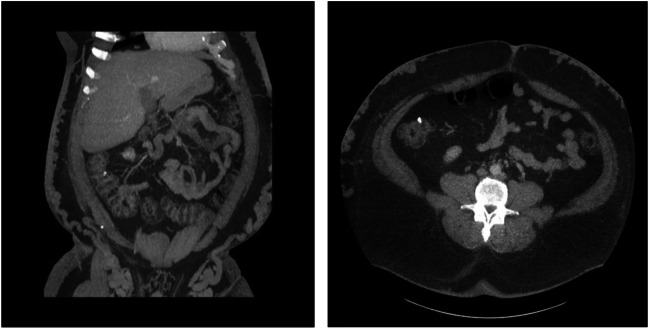
CT demonstrating venous collaterals arising from the common iliac veins on both sides and running along the flanks to the azygos and just below the skin in the subcutaneous plane

He had a CT scan which revealed numerous venous collaterals in the anterior and lateral abdominal wall in the subcutaneous plane distributed symmetrically, and this appeared to originate from both the groins. The IVC was small in calibre measuring 1.7 cm in maximal axial dimension and appeared to be atretic. There was no evidence of IVC thrombosis (Fig. 1).

**Fig. 2:**
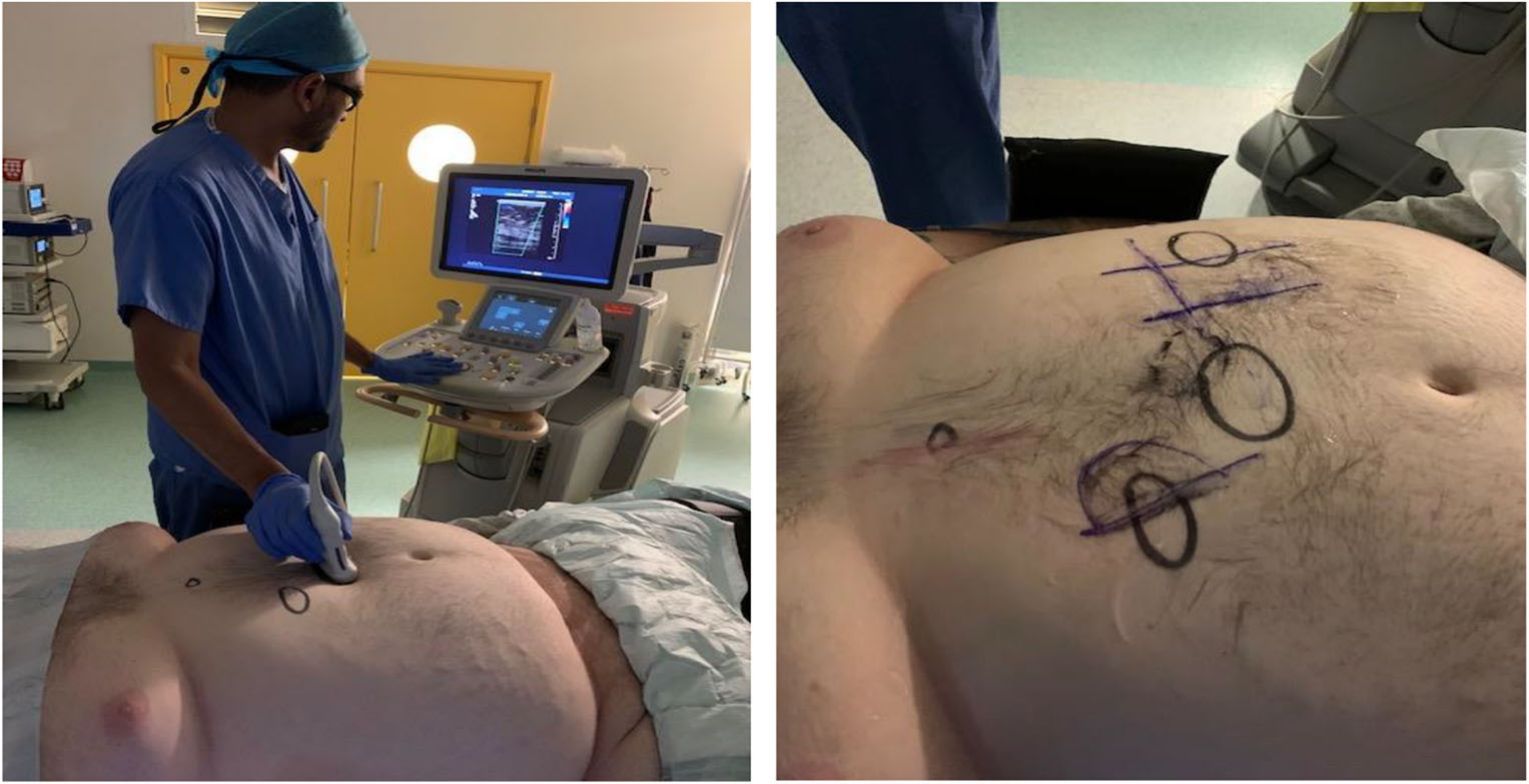
Pre-op ultrasound-guided marking of port sites prior to laparoscopic sleeve gastrectomy

The patient was counselled for laparoscopic sleeve gastrectomy. He underwent on table preoperative US-guided mapping of the anterior abdominal wall to guide port placement with a view to avoid injury to the venous collaterals (Fig. 2). The port sites were slightly deviated from the standard port positions, and the patient underwent standard laparoscopic sleeve gastrectomy. He made a good recovery and is doing well on post-op follow-up.

## Discussion

Congenital anomalies of the IVC are not uncommon with significant variations due to the complexities around its development during embryogenesis [1]. Awareness of these anomalies is important to avoid pitfalls in management of these patients as they are mostly asymptomatic.

One of the rare presentations of patients with congenital anomalies of the IVC is DVT at a young age [2]. A recognized variant is the absence or atretic infra-renal IVC as is the case in our patient. This results in development of collaterals from the iliac veins draining along the abdominal wall into azygos and hemiazygos vein. There is controversy as to whether this is a true embryonic anomaly or whether it is the result of perinatal IVC thrombosis [3].

It is suggested that the venous return in the case of an absent or atretic IVC is suboptimal in spite of collaterals and hence results in chronic venous hypertension in the lower limbs leading to chronic venous insufficiency and venous ulcers as was the case in our patient.

CT is a very reliable noninvasive imaging modality in diagnosing IVC anomalies. This rules out other causes of distended venous collaterals in the abdominal wall, especially those associated with portal HT in the background of NASH [4].

This is the first reported case in literature where any form of laparoscopic surgery has been done in a patient with engorged blood vessels in the anterior abdominal wall due to atretic IVC. This is certainly the first patient with morbid obesity who had laparoscopic sleeve gastrectomy. With preoperative ultrasound marking, the surgery can be done in a safe manner as has been demonstrated in our patient.

## Conclusion

Congenital malformations of the IVC are rare. Most patients are asymptomatic, and the anomalies are picked up during radiological investigations. Knowledge of these congenital anomalies is important prior to performing any operative/laparoscopic surgery and especially in patients with obesity and associated NASH as it could be potentially labelled as part of the picture of portal HT and the patients being denied bariatric surgery. Preoperative ultrasound-guided placement of ports enables any laparoscopic procedure including bariatric surgery to be done safely as has been demonstrated in this patient.

## Abbreviations

AF: Atrial fibrillation
BMI: Body mass index
CT: Computed tomography
DVT: Deep vein thrombosis
HT: Hypertension
IVC: Inferior vena cava
NASH: Non-alcoholic Steatohepatitis
NIDDM: Non-insulin-dependent diabetes mellitus
OSA: Obstructive sleep apnea
PV: Portal vein
USS: Ultrasound scan

## References

[1] Edward Bass J, Redwine MD, Kramer LA, et al. Spectrum of congenital anomalies of the inferior vena cava : Cross sectional imaging findings. Radiographics. 2000:20(3). 10.1148/radiographics.20.3.g00ma09639. 10.1148/radiographics.20.3.g00ma09639 10835118

[2] Chee YL, Dominic J, Watson CG, Watson HG (2001). Inferior vena cava malformation as a risk factor for deep venous thrombosis in the young. Br J Haematol.

[3] Tharumenthiran R, Hughes TMD, Richardson AJ (2001). Perinatal inferior vena cava thrombosis and absence of the infrarenal inferior venacava. J Vasc Surg.

[4] Ueda J, Hara K, Kobayashi Y, Ohue S, Udrida H (1983). Anomaly of the inferior vena cava observed by CT. Comput Radiol.

